# Profile of health facilities and determinants of the family planning services supply to unmarried adolescents in Burkina Faso, Ghana, and Niger

**DOI:** 10.4314/gmj.v56i3s.13

**Published:** 2022-09

**Authors:** Nassirou Ibrahim, Roxane Borgès Da Silva

**Affiliations:** University of Montreal School of Public Health (ESPUM)

**Keywords:** Family planning services package, adolescents, health facilities, GEE model

## Abstract

**Objectives:**

Despite numerous interventions to facilitate adolescents' access to family planning (FP) services in West Africa, studies reveal that unmarried adolescents have difficulties accessing these services. This study analyses the supply of the FP services package to unmarried adolescents as well as the profiles of the facilities that provide this package in Burkina Faso, Ghana, and Niger. Also, it examines the determinants of the supply of this package.

**Design:**

The study adopted a spatiotemporal descriptive analysis and a binary logistic Generalized Estimating Equation (GEE) model. The data come from surveys conducted in the three countries between 2013 and 2019 as part of the Performance Monitoring and Accountability 2020 program.

**Participants:**

The target population consists of health facilities that provide health services.

**Results:**

The study indicates that more than 80% of FP services are provided by basic health facilities in Burkina Faso and Niger, while in Ghana, the profile is more diversified, including hospitals, polyclinics, and public and private primary health centres. The econometric analysis indicates that regional ownership, examination of client opinion data, ownership of a functioning computer, and knowledge of the served population are the main determinants of the supply of the FP services package to unmarried adolescents.

**Conclusion:**

By identifying facility profiles and determinants of FP services supply, this study provides a pathway for action to ensure that adolescents have access to these services regardless of their marital status in West Africa.

**Funding:**

This work was carried out with the support of the COMCAHPSS project funded by IDRC and the Adolescent Health in West Africa (Adowa) project funded by MRC

## Introduction

Since the International Conference on Population and Development (Cairo, 1994), the provision of sexual and reproductive health (SRH) and family planning (FP) services has been recognised as a necessity for improving the health and well-being of adolescents.^1^–^3^ Public and private health facilities in several developing countries were supported to offer available and adequate adolescent SRH and FP services. For example, the “*Adolescent Friendly Services”* program was designed and implemented in Burkina Faso, Ghana, and Niger in 2000 to use public and private health facilities as a channel to make SRH services, and specifically FP services, available and accessible to adolescents. The FP service package included mainly counselling on FP methods, referral to specialised FP facilities, prescriptions, and provision of modern contraceptive methods to adolescents. 1–4 In Niger, for example, since 2006, the government, with support from partners such as UNFPA, has labelled more than 40 public health facilities as “adolescent-friendly health facilities”.

These facilities were equipped with staff trained in adolescent-specific reproductive health issues and in providing appropriate services and commodities.^3^ These efforts have also been observed in Burkina Faso and Ghana.

Despite numerous efforts, however, recent indicators show that adolescents still have very limited access to FP services.^3^,^5^,^6^ This raises the question of whether FP services are available and accessible to all categories of adolescents. Indeed, studies reveal that in reality many health facilities do not offer FP services to unmarried adolescents, thereby depriving them of the same access to services as their married counterparts.^3^,^7^ In a context where all countries aim to achieve universal health coverage by 2030 through the Sustainable Development Goals (SDGs) agenda, this situation deserves special attention. But, to act adequately, evidence on adolescent FP services access is needed.

The objective of this study is therefore to generate evidence on adolescents' access to FP services. Specifically, it aims to analyse the supply of the FP services to unmarried adolescents, to identify the profile of health facilities offering FP services in Burkina Faso, Ghana, and Niger, and to analyse the determinants of the package supply in the three countries. The countries were chosen by the need to reflect the two main official languages of the sub-region (English and French) and related contextual variations in the study. Socio-demographic and health indicators also guided the selection of countries. Niger has more critical indicators, Ghana has better indicators and a more advanced health system, and Burkina Faso has a relatively intermediate situation compared to the other two countries.^8^–^10^ For example, data indicate a 6% contraceptive prevalence rate among 15–19 year-olds in Niger compared to 15% in Ghana and 13% in Burkina Faso.^9^ Similarly, the proportion of sexually active adolescents aged 15–19 years is 38% in Niger and 25% in Burkina Faso while it is only 14% in Ghana.^9^,^11^,^12^

## Methods

### Design and Data sources

This was a cross-sectional study that used data from the Performance Monitoring and Accountability 2020 (PMA 2020) surveys. These surveys, cross-sectional in nature with repeated rounds every six months, are part of a data collection project to capture and monitor the level of FP indicators in 11 developing countries, including Burkina Faso, Ghana, and Niger. For reasons of data availability and comparison of results across countries, we used data from rounds 1, 2, 3, 4, 5, and 6 for Burkina Faso, data from rounds 1, 3, 4, 5, and 6 for Ghana, and data from rounds 2 and 4 for Niger (Table 1).

**Table 1:** Samples size by country and year

Round	Burkina Faso (bf)		Ghana (gh)		Niger (ne)		The three countries	
	Year (t)		Year (t)		Year (t)		Year (t)	*n*_tg_*
**Round 1**	2014	107	2013	143			2016	438
**Round 2**	2015	107			2016	135		
**Round 3**	2016	134	2014	241				
**Round 4**	2017	133	2015	239	2017	132	2017	464
**Round 5**	2018	133	2016	169				
**Round 6**	2019	101	2017	199				

**Source**: Data from PMA2020 surveys (Burkina Faso, Ghana, and Niger, 2013–2019).

**Note:** * It means the three countries together.

### Target Population and Samples

The target population is constituted of public and private health facilities that provide health services to the population. Table 1 presents the samples by country and year.

### Ethical clearance

PMA2020 collects data in accordance with the ethical principles of each country. Free and informed participant consent is obtained before the interview. The data are de-identified and available online. Permission has been obtained from PMA2020 to use the data for this work. In addition, this study is part of doctoral thesis work at the University of Montreal's School of Public Health. The research protocol has been approved by the University of Montreal's Health and Science Research Ethics Committee (CERSES) (CERSES-20-128-P) and the National Health Research Ethics Committee of Niger for the January 28, 2021, session.

### Variables

#### Dependent variable

The dependent variable is “FP services package supply to unmarried adolescents.” It is constructed from the combination of three binary variables: (i) offering counselling on FP methods to unmarried adolescents (1=Yes and 0=No) (ii) offering modern FP methods to unmarried adolescents (1=Yes and 0=No), and (iii) prescribing contraceptive methods to adolescents or referring adolescents to other more specialised FP facilities (1=Yes and 0=No). After treatment, the dependent variable was transformed into a compound binary variable. The transformation was done according to the following conditions: when the health facility counsels unmarried adolescents on FP methods, offers FP methods to unmarried adolescents, and prescribes contraceptive methods to unmarried adolescents (or refers them to other more specialised FP facilities), the variable took the value 1. If one of the three conditions was not satisfied, the variable took the value 0. The distribution of health facilities offering or not each type of PF service to unmarried adolescents by model is showed in the Table 2.

**Table 2:** Distribution of health facilities that provide or not PF services to unmarried adolescents by type of service

	Model 1: The three countries							
	Counselling on family planning methods to unmarried adolescents		Provided family planning methods to unmarried adolescents		Prescribed/referred family planning methods to unmarried adolescents		Supply of PF services Package *(discrete composite* *variable)*	
	Number	%	Number	%	Number	%	Number	%
**Yes**	64	7%	85	9%	362	40%	468	52%
**No**	755	84%	734	81%	457	51%	434	48%
**Total**	902	100%	902	100%	902	100%	902	100%
	**Model 2: Burkina Faso**							
	Number	%	Number	%	Number	%	Number	%
**Yes**	27	4%	49	7%	192	27%	264	37%
**No**	688	96%	666	93%	523	73%	451	63%
**Total**	715	100%	715	100%	715	100%	715	100%
	**Model 3: Ghana**							
	Number	%	Number	%	Number	%	Number	%
**Yes**	80	8%	64	6%	330	33%	450	45%
**No**	911	92%	927	94%	661	67%	541	55%
**Total**	991	100%	991	100%	991	100%	991	100%
	**Model 4: Niger**							
	Number	%	Number	%	Number	%	Number	%
**Yes**	38	14%	41	15%	153	57%	187	70%
**No**	229	86%	226	85%	114	43%	80	30%
**Total**	267	100%	267	100%	267	100%	267	100%

**Source:** Data from PMA2020 surveys (Burkina Faso, Ghana, and Niger, 2013–2019).

#### Independent variables

The independent variables (see Table 3) were selected based on the literature review 2,3,13–15 examining the supply and utilisation of health services, especially PF services.

**Table 3:** Description of independent variables by country and model

Variable	Description			
	Model 1 (Mo.1): *The three countries*	Model 2 (Mo.2): *Burkina Faso*	Model 3 (Mo.3): *Ghana*	Model 4 (Mo.4): *Niger*
**Country of residence**	1. Burkina Faso 2. Ghana 3. Niger			
**Region of residence**		1. West 2. Northeast 3. Centre	1. North 2. East-Central 3. West	Niamey-Tillabéri 2. Dosso-Tahoua-Agadez 3. Maradi-Zinder-Diffa
**Place of residence**	1. Urban 0. Rural	1. Urban 0. Rural	1. Urban 0. Rural	1. Urban 0. Rural
**Type of health facility**		1. MCSU 2. HSPC 3. Private-other	1. hospital/polyclinic 2. health centre 3.healthclinic_CHPS_Chemist_other	1. IHC 2. health care facility 3. Private _other
**Managing Authority**	*1. Government* *0. Private*	*1. Government* *0. Private*	*1. Government* *0. Private*	*1. Government* *0. Private*
**Number of days booked for** **FP services**	1. 5 days 2. 6 days 3.7 days	1. 5 days 2. 6 days 3. 7 days	1. 5 days 2. 6 days 3. 7 days	1. 5 days 0. 6 or 7 days
**Review of client opinion** **data**	1. yes 0. No	1. yes 0. No	1. yes 0. No	1. yes 0. No
**Number of beds available**	number	number	number	
**Billing for contraceptive** **methods**	1. yes 0. No	1. yes 0. No	1. yes 0. No	1. yes 0. No
**Existence of a working computer**	1. yes 0. No	1. yes 0. No	1. yes 0. No	1. yes 0. No
**Knowledge of the population** **served**	1. yes 0. No	1. yes 0. No	1. yes 0. No	1. yes 0. No
**Condition of the FP services** **room**	1. Clean 0. In poor condition	1. Clean 0. In poor condition	1. Clean 0. In poor condition	1. Clean 0. In poor condition
**Year**	1. 2016 0. 2017	1. 2014 2. 2015 3. 2016 4. 2017 5. 2018 6. 2019	1. 2013 2. 2014 3. 2015 4. 2016 5. 2017	1. 2016 0. 2017

**Source:** Data from PMA2020 surveys (Burkina Faso, Ghana, and Niger, 2013–2019).

**Notes (Table 2)**

**Modality/acronyms**   **Grouping/definition**

**Region of residence (Burkina Faso)**

West     Boucle du Mouhoun, Cascades, Hauts-Bassins and South-West

Northeast     North Central, North, East and Sahel

Centre     Central, East Central, South Central, West Central

**Region of residence (Ghana)**

Northern-Upper_East-West     Northern, Upper-east and Upper-west

East-central     Central, Eastern, Volta and Greater-Accra

West     Western, Ashanti and Brong-Ahafo

IHC     Integrated Health Centre

MCSU     Medical centre with surgical unit

HSPC     Health and Social Promotion Centre

CHPS     Community-based Health Planning and Services

### Model and analysis methods

#### Model specification

A binary logistic regression model was chosen to model the dependent variable. This choice was based firstly on the nature of this variable and secondly on the possibilities that this type of model offers to estimate and interpret the results.^16^–^18^ Also, it allows “extreme” events to be assigned a higher probability than the normal distribution.

Modelling the supply of the FP services package to un-married adolescents (**Y**) was based on the notion of a latent variable.^16^–^18^ This assumes the existence of an un-observable variable (Y*) which is a function of the characteristics of the health facility and its environment.

Thus, for a health facility i that has offered the package of FP services to unmarried adolescents at date t, we have:


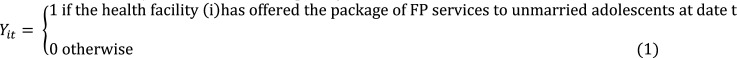




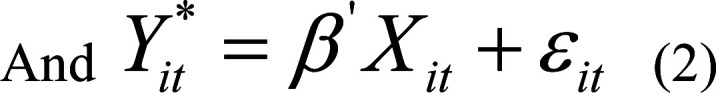



Where X_*it*_ (1, x_1*it*_, x_2*it*_, ..., x_nit_) is a vector of n independent variables (see Table 3), *β*(*β*_0_,*β*_1_,...,*β*_n_) is a vector of parameters to be estimated and *ε_it_*is the error term.

Defining by *P_it_* the probability that a health facility i offers the package of FP services to unmarried adolescents at date t, we have:


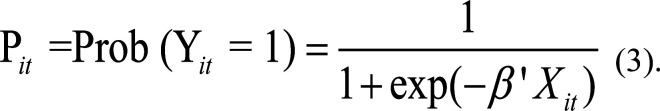



### Methods of analysis

First, we conducted a trend and cross-country analysis to analyse the supply of the FP services package to unmarried adolescents and to identify the profile of the health facilities that provide it (objectives 1 and 2). Second, we used the Generalized Estimating Equations (GEE) model 19,20 to examine the determinants of FP services supply to unmarried adolescents (objective 3). The choice of this model is justified by the fact that it considers the intra-subject correlation, which, if not considered, leads to invalid statistical inferences. The intra-subject correlations are considered by the specification of a correlation structure, “*A working correlation* structure”^21^, allowing sample population-level results.

In total, four models were estimated. One model for each of Burkina Faso, Ghana, and Niger and one model for all three countries together. The empirical validation of these models was carried out using the global validity test (Wald Khideux test) at the 5% threshold and using the quasi-likelihood under the Independence model Criterion (QIC) with different correlation matrices.^21^–^23^ After numerous simulations, we selected the GEE models with a “compound symmetry” correlation matrix with the lowest QIC values.^21^–^23^ The Odds Ratio (OR) is used to interpret the results of analysis produced using Stata software.

## Results

The results of this study focus on three aspects: the trend in the supply of the FP services package to unmarried adolescents, the profile of the health facilities that provide the package, and the factors that explain its supply.

### Trends in the supply of the FP services package to un-married adolescents

Trend analyses indicate that in 2017, just 29% (compared to 33% in 2016) of health facilities offered the package in Niger. This proportion is higher in Ghana and Burkina Faso, for both years, with Burkina Faso having the highest proportions across all the years surveyed. (Table 4).

**Table 4:** Proportion (%) of facilities that offered the FP services package to unmarried adolescents by year and country

Year	Burkina Faso		Ghana		Niger	
	Number	%	Number	%	Number	%
**2013**			143	46.94		
**2014**	107	74.22	241	57.84		
**2015**	107	67.58	239	51.01		
**2016**	134	57.53	169	50.02	135	33.46
**2017**	133	70.38	199	54.30	132	29.29
**2018**	133	71.54				
**2019**	101	64.87				

**Source:** Data from PMA2020 surveys (Burkina Faso, Ghana, and Niger, 2013–2019).

### Profile of health facilities offering the FP services package to unmarried adolescents

**Figure 1:**
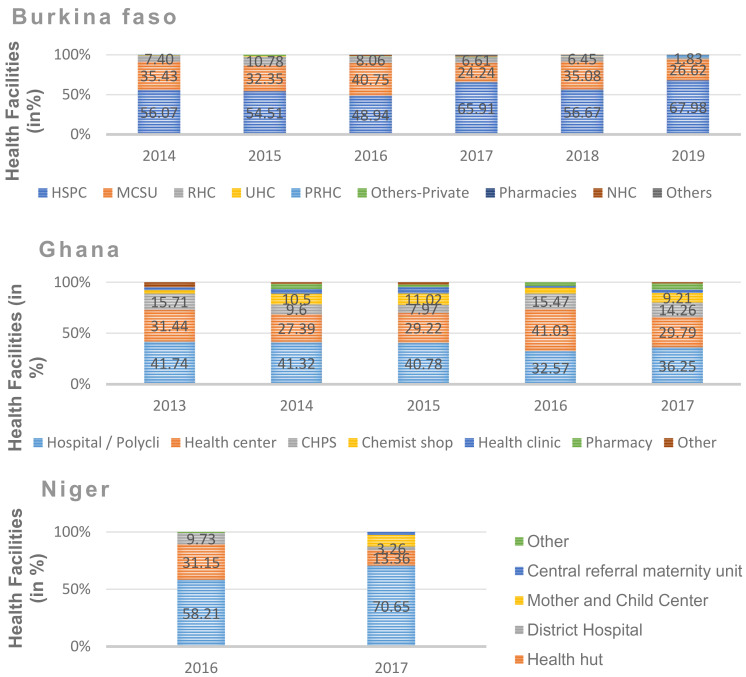
Proportion (in %) of health facilities that offered the FP services package to unmarried adolescents between 2013 and 2019 by country Notes (Figure 1) Acronyms      Expression CSPS     Health and Social Promotion Centre MCSU     Medical centre with surgical unit RHC     Regional Hospital Centre UHC     University Hospital Centre PRHC     Private Health Centre HPCCP     Hospital/polyclinic/private clinic NHC     National Hospital Central

Country level data analysis indicates that in Niger, integrated health centres (IHCs) and health huts (HHs) offer the most FP services to unmarried adolescents. In 2017 for example, out of 100 health facilities offering the FP package to unmarried adolescents, 71 were IHCs and 13 were HHs (see Figure 1). This predominance was also observed in 2016 although to a lesser extent. Beyond these health centres, private health facilities such as pharmacies also offered FP services, but in marginal proportions (0.9% in 2016).

In Burkina Faso, the same pattern emerged. In 2019, the data indicated that more than 89% of the facilities offering FP services to unmarried adolescents are social centres for health promotion, including health and social promotion centres (CSPS) (68%) and medical centres with a surgical unit (MCSU) (27%) (see Figure 1). The private sector is also represented, as in Niger, with a marginal proportion (0.6% in 2016). A comparative analysis shows that the FP service is offered to unmarried adolescents in Burkina Faso in some regional hospitals (8.62% in 2016), whereas in Niger, the package is rather offered in district hospitals (9.73% in 2016)

Unlike Burkina Faso and Niger, in Ghana, the profile of health facilities offering the FP services package to unmarried adolescents is more differentiated and varied. Hospitals and polyclinics appear to dominate in providing this package. In 2017, for example, of the facilities offering FP services to unmarried adolescents, 36% were hospitals and polyclinics, 29% were health centres, 12% were *Community-based Health Planning and Service”* (CHPS), 8% were chemist shops, and 4% were pharmacies (see Figure 1). For 2013, 2014 and 2016, the same dominance is observed in favour of hospitals and poly-clinics. However, in 2016 health centres recorded the largest proportion (41%) in supplying the FP services package to unmarried adolescents, followed by hospitals and polyclinics (33%).

### Determinants of FP services package supply to un-married adolescents

Factors related to the context of residence, the characteristics of health facilities, and time determine the availability of the FP services package among unmarried adolescents (Table 5).

**Table 5:** Odds ratios (ORs) of the likelihood of offering the package of FP services to unmarried adolescents.

Variables/Modalities	Mo.1 (Set)		Mo. 2 (Burkina Faso)		Mo. 3 (Ghana)		Mo. 4 (Niger)	
	OR	[95 % CI]	OR	[95 % CI]	OR	[95 % CI]	OR	[95 % CI]
**Country of residence**								
***Burkina Faso***	Ref.							
***Ghana***	1. 064***	[0.689–1.642]						
***Niger***	0.368	[0.245–0.553]						
**Region of residence**								
**Boucle du Mouhoun, Cascades,** **Hauts-Bassins and** **South-West**			Ref.					
**North Central, North, East,** **and Sahel**			2. 911***	[1.523–5.565]				
**Central, East Central, South** **Central, West Central**			0.884	[0.523–1.494]				
**Northern, Upper-east and Upper-west**					Ref.			
**Central, Eastern, volta and** **Greater-Accra**						0.833	[0.530–1.309]	
**Western, Ashanti and Brong-Ahafo**					0.554***	[0.353–0.871]		
***Niamey-Tillabéri***							Ref.	
***Dosso-Tahoua-Agadez***							1. 139	[0.552–2.351]
***Maradi-Zinder-Diffa***							2. 264**	[1.087–4.714]
**Place of residence**								
***Rural***	Ref.							
***Urban***	1. 317*	[0.954–1.818]	1. 281	[0.800–2.051]	1.36*	[0.973–1.918]	0.972	[0.492–1.920]
**Type of health facility**								
***Medical centres with surgical*** ***units (MCSU)***			Ref.					
***Health and social promotion*** ***centres (HSPC)***			1.661**	[1.059–2.606]				
***Private-other***			0.689	[0.286–1.658]				
***Hospital-polyclinic***					Ref.			
***Health Centre***					1.582**	[1.029–2.431]		
***Health clinic-CHPS-Chemist-other***					1.550*	[0.936–2.567]		
***Integrated Health Centres*** ***(IHC)***							Ref.	
***Health hut***							0.842	[0.311–2.277]
***Private-other***							0.483	[0.176–1.324]
**Managing Authority**								
***Private***	Ref.							
***Government***	1. 787**	[1.006–3.175]	12.9***	[3.216–49.521]	2. 067***	[1.241–3.444]	3. 076	[0.078–122.096]
**Number of days booked for** **FP services**								
***5 days***	Ref.							
***6 days***	1. 316***	[0.764–2.268]	3. 268*	[0.983–10.863]	1. 272	[0.827–1.957]	1. 089	[0.518–2.289]
***7 days***	1. 773	[1.232–2.551]	1. 012	[0.544–1.885]	1. 791***	[1.214–2.641]		
**Review of client opinion data**								
***No***	Ref.							
***Yes***	3. 596***	[1.960–6.597]	1. 754*	[0.885–3.475]	1. 892***	1.219–2.939]	4. 036***	[1.631–9.985]
**Number of beds available**			1. 000	[0.999–1.0009]	1.0001***	[1.0006–1.0001]	0.994	[0.985–1.003]
**Billing for contraceptive** **methods**								
***No***	Ref.							
***Yes***	1. 022***	[1.006–1.039]	0.975	[0.946–1.006]	3. 161***	[1.922–5.198]		
**Existence of a working computer**								
***No***								
***Yes***	1. 534	[0.905–2.600]	2. 285***	[1.283–4.070]	1. 463*	[0.934–2.293]	2. 974***	[1.032–8.568]
**Knowledge of the served population**								
***No***	Ref.							
***Yes***	0.929	[0.621–1.390]	2. 347**	[1.092–5.046]	1. 406*	[0.942–2.097]	0.560	[0.250–1.252]
**Condition of FP services room**								
***In poor condition***	Ref.							
***Clean***	4. 412***	[2.628–7.407]	1. 383	[0.297–6.433]	1. 480	[0.818–2.678]	5. 418***	[1.629–18.016]
**Year (time effect)**								
***2013***					Ref.			
***2014***			Ref.		2. 568***	[1.585–4.162]		
***2015***			0.673	[0.367–1.234]	2. 271***	[1.416–3.641]		
***2016***	Ref.		0.397**	[0.222–0.710]	5. 618***	[2.567–12.296]	Ref.	
***2017***	0.881	[0.616–1.260]	1. 200	[0.264–6.354]	4. 939***	[2.287–10.666]	0.457	[0.132–1.577]
***2018***			1. 700	[0.732–3.945]				
***2019***			1. 031	[0.378–2.807]				
**Constant**	0.3902**	[0.160–0.950]	0.006***	[0.0005–0.066]	0.047***	[0.017–0.129]	0.075**	[0.008–0.711]
**Observation (N)**	**902**		**715**		**991**		**267**	
**Number of periods (T)**	**2**		**6**		**5**		**2**	
**Number of groups (K)**	**542**		**226**		**425**		**197**	
**Wald chi2 ([n-k] degree of** **freedom)**	**111.62 (13)**		**112.16 (20)**		**184.80 (19)**		**35.12 (14)**	
**Prob > chi2 =**	**0.0000**		**0.0000**		**0.0000**		**0.0014**	
**QIC (with compound symmetry** **as matrix structure)**	**1,122. 830**		**776. 598**		**1,209. 827**		**314. 038**	

**Source:** Data from PMA2020 surveys (Burkina Faso, Ghana, and Niger, 2013–2019).

**Note:** ***, **, * respectively the significance of the values of the odds ratios, CI: Confidence Interval.

**Modality/Acronyms**     **Grouping/definition**

West     Boucle du Mouhoun, Cascades, Hauts-Bassins and South-West

Northeast     North Central, North, East, and Sahel

Centre     Central, East Central, South Central, West Central

Northern-Upper_East-West     Northern, Upper-east and Upper-west

East-central     Central, Eastern, volta and Greater-Accra

West     Western, Ashanti and Brong-Ahafo

CHPS     Community-based Health Planning and Services

### Country and Place of Residence

The results indicate that health facilities in Ghana are 1.06 times more likely to offer the FP services package to unmarried adolescents than those in Burkina Faso. In contrast, there was no significant difference between the latter and Niger. Although residence appears to be an un-observed factor in the overall model (Mo.1) and countries such as Burkina Faso (Mo.2) and Niger (Mo.4), in Ghana, it has a strong influence at the 5% threshold. As presented in Table 4, health facilities in urban areas of Ghana are 1.36 times more likely to offer FP services to unmarried adolescents than those in rural areas.

### Region of Residence

Region of residence was an important determinant of the FP services supply to unmarried adolescents in all three countries. In Burkina Faso, for example, although there is no significant difference at the 5% threshold between the “Centre-North, North, East and Sahel” regions and the “South-West, Cascades-High, Basins and South-East” regions, facilities in the “Centre, Centre-East, Centre-South and Centre-West” regions were thrice more likely to offer the FP services package to unmarried adolescents than those in the “South-West, Cascades-High, Basins and South-East” regions. On the other hand, in Ghana, facilities in the Ashanti, Western and Brong-Ahafo regions are 45% less likely to offer FP services to unmarried adolescents than those in the Northern, upper-east, and upper-west regions. However, there was no significant difference between the latter and the health facilities in the Central, Eastern, Volta and Greater-Accra regions. As in Burkina Faso and Ghana, regional differences emerge in Niger. Health facilities in the Maradi-Zinder-Diffa regions are 2.3 times more likely to offer FP services to unmarried adolescents than those in the Niamey-Tillabéri regions.

### Characteristics of Health Facilities

Health facilities' many characteristics determine the FP services supply to unmarried adolescents in the three countries (Table 5). The most salient characteristics are presented below.

### Type of Facilities

In Burkina Faso, Health, and Social Promotion Centres (HSPC) are 1.7 times more likely to offer the FP services package to unmarried adolescents than Medical Centres with Surgical Units (MCSU). In Ghana, on the other hand, health centres are more likely to offer FP services to unmarried adolescents, as they are 1.6 times more likely to offer FP services than hospitals and polyclinics. The odds ratio is slightly lower for clinics and community-based health planning and services, which have an odds ratio of 1.5. Furthermore, the type of facility does not influence the supply of FP services in Niger. The same is true for the “management authority” variable, but with the difference that in Burkina Faso and Ghana, the Government-run facilities are 12.6 and 2.1 times more likely to supply the package of FP services to unmarried adolescents than privately run facilities.

### Review of Client Opinion Data

Overall model results indicate that facilities examining client opinions are 3.6 times more likely to offer the FP services package to unmarried adolescents than those that do not examine client opinions. The odds ratio is 1.8 for facilities in Burkina Faso, 1.9 for facilities in Ghana, and 4.0 for facilities in Niger.

### Knowledge of the Served Population

In the global and Niger models, no significant difference is observed between health facilities knowing or not knowing their served population. However, differences are observed in Burkina Faso and Ghana. In Burkina Faso, for example, facilities that know their served population are 2.3 times more likely to offer FP services to unmarried adolescents than those that do not know their served population. In Ghana, this odds ratio is 1.4 for facilities that know their served population.

### Billing for FP Services

In the overall model, facilities that charge for FP services are more likely to offer the FP services package to un-married adolescents than those that do not charge for the same services. In Ghana, this odds ratio is much higher (Table 5)

### Condition of the FP Services Room

Some studies show that cleanliness plays an important role in the supply of health services, particularly reproductive health services. Results indicate that facilities with a clean FP service room are 4.4 times more likely to supply FP services to unmarried adolescents than those with a poor or non-existent room. This odds ratio is 5.4 for facilities in Niger with a clean FP room.

### Time Effect

In addition, time dynamics also play an important role in determining FP services package supply to unmarried adolescents. Nevertheless, the time effect is very nuanced from one model to another. For example, for the overall and Niger models, the time effect is insignificant at the 5% threshold. This result could be explained by the fact that the data used to estimate the two models are limited only to two years: 2016 and 2017. In contrast, the Burkina Faso and Ghana models used data for more than three years, and the time effect is highly significant, especially in Ghana.

## Discussion

To better ensure accessibility and availability of SRH services among adolescents, countries such as Burkina Faso, Ghana, and Niger have, since the 2000s, integrated FP services among the essential health services for adolescents regardless of their marital status. However, the results of this study indicate that in countries like Niger, very few (30%) health facilities offer these services. If all adolescents should have access to essential services to achieve universal health coverage, this situation raises questions about the appropriateness of interventions in the country's socio-cultural context. 24 The results also show a heterogeneous profile of the structures that offer these services from one country to another. In Ghana, for example, we find a more diversified profile composed of health centres, hospitals, polyclinics and CHPS, while in Burkina Faso and Niger, third-level or peripheral health facilities offer services. Also, in Ghana, the private sector is quite present, whereas it is only in a very marginal proportion (less than 1%) in the other countries. This situation is less surprising given that the organisation and management of the health system in each country are different, despite all three target countries being members of the Community of West African States (ECOWAS) and its specialised West African Health Organisation (WAHO) that supports the development of guidelines and strategies to enable countries to organise better and manage their health care systems.^11^ Ghana's advanced level of implementation of FP interventions may also explain the differences.

The econometric analysis indicates that many factors contribute to determining the supply of the FP services package to unmarried adolescents. Indeed, the results show that urban rather than rural health facilities, especially in Ghana, are more likely to supply the FP services package to unmarried adolescents. This could be explained by the fact that the supply of FP services to adolescents generally requires skilled personnel who are very limited in rural areas.^24^ Also, socio-cultural norms and values have more influence on people's behaviour in rural areas than in urban areas, which are more exposed to modernity.^7^,^25^

It was also found that health facilities that charge for FP services provide more FP services to unmarried adolescents than those that do not. This result can be explained by the fact that FP services are considered a marketable or private good and offering them can benefit health facilities. However, considering these services as a private good can limit their demand because, according to economic theory, a private good obeys the principle of exclusion, i.e., adolescents who do not have the means to have access will be automatically excluded from these services despite showing a need.^14^,^26^,^27^

The study also reveals that knowledge of the served population, examining data on client opinions, and time explain the supply of FP services packages to unmarried adolescents. Indeed, for authors like Pineault^13^, offering health services to a population requires a prior assessment of their needs. Undeniably, this requires questioning socio-demographic characteristics including age, sex, and marital status. Therefore, a health facility that knows the characteristics of its served population will be more likely to offer services that meet the needs of the different groups than one that lacks knowledge.

As part of a *“patient-partner”* approach 28, examining clients' opinions appears in several studies to be a determining factor in improving the supply of health services to the population. Any health facility engaging in such approaches seeks to collect useful information to better understand its clients' needs and respond adequately. Since the channels (suggestion box, anonymous letter, telephone number) for collecting opinions are generally discrete and confidential, clients, especially unmarried adolescents, can easily use them to express their FP needs. This allows the health facility to integrate the adolescents' PF needs into the services delivered .^28^

The results show that the supply of the FP services package to unmarried adolescents has evolved. Based on the multiple policies and programs implemented in favour of adolescent sexual and reproductive health in Burkina Faso, Ghana, and Niger since 2000, the expected effect should be an increasing FP services supply to adolescents, regardless of their marital status. However, the results indicate both increasing and decreasing progress. This indicates inertia in the behaviour change of actors providing FP services, especially to unmarried adolescents. In other words, some health facilities or providers are still under the influence of certain coercive socio-cultural norms that consider using FP services, including contraceptive methods by unmarried adolescents, as a depravity of social norms.^2^,^3^

### Limitations

Despite the study's interesting results, there are some limitations. Many contextual and individual factors are not considered because they are unavailable in the database. These include certain health facility socio-demographic characteristics, such as the number of years of experience, the gender of the head of facilities, the source of supply of FP products and the number of staff available. Also, we note that using data from different control cycles and countries may affect the comparability of the results between countries by introducing biases related to country structures and the time lag of the observed data. Nevertheless, the use of the same sampling and data collection methods in the three countries and the fact that our comparative analyses were conducted on data collected in the same years would reduce these biases.

Another limitation is that the study is based on surveys and self-reported data. There may therefore be a desirability bias. Thus, further in-depth qualitative and quantitative studies are needed to examine the supply of the FP services package to married and unmarried adolescents in West Africa.

## Conclusion

Since the 2000s, countries such as Burkina Faso, Ghana and Niger have implemented many interventions to improve adolescent sexual and reproductive health. These countries have instituted FP services for adolescents regardless of their marital status, and several health facilities have been responsible for providing them. However, many adolescents, especially unmarried adolescents, have difficulty accessing PF services.^1^–^3^ This study sought to generate evidence to understand better the problems these adolescents encountered in accessing PF services. Results indicate that unmarried adolescents face difficulties due to the unavailability of these FP services in some public and private health facilities. In Niger, only 30% of health facilities offered the FP services package to unmarried adolescents in 2017. This proportion was about 54% in Ghana and 70% in Burkina Faso in 2017. Also, this study identified many factors that explain the supply of FP services to adolescents. The most salient is the place of residence, region of residence, examination of data on client opinions, billing for contraceptive methods, knowledge of the served population and possession of a functional computer. These factors can thus serve as benchmarks for future interventions in Burkina Faso, Ghana, and Niger.
